# When the Treatment Becomes the Threat: A Case of Rituximab-Induced Organizing Pneumonia

**DOI:** 10.7759/cureus.117468

**Published:** 2026-10-05

**Authors:** Bernardo Belchior, Tiago Jorge Costa, Ana Saraiva, Sofia Brasil, Joana Paixão

**Affiliations:** 1 Internal Medicine, Coimbra Hospital and University Centre, Coimbra, PRT

**Keywords:** corticosteroids, drug-induced interstitial lung disease, non-hodgkin lymphoma, organizing pneumonia, rituximab

## Abstract

Rituximab (RTX) is a chimeric monoclonal antibody against CD20-expressing B lymphocytes, widely used in B-cell malignancies. Although generally well-tolerated, pulmonary toxicity is a rare but potentially fatal complication. A 71-year-old male with hepatic non-Hodgkin lymphoma (NHL) in remission, on maintenance RTX, presented with a one-month history of fever, asthenia, and progressive dyspnea. Initial management for suspected community-acquired pneumonia with levofloxacin failed. Despite temporary improvement on piperacillin/tazobactam, symptoms recurred after discharge, progressing to hypoxemic respiratory failure. Extensive workup, including bronchoalveolar lavage (BAL) and positron emission tomography-computed tomography (PET-CT), ruled out infection and lymphoma relapse. Following a multidisciplinary review, RTX-induced organizing pneumonia (OP) was suspected. Initiation of high-dose methylprednisolone (250 mg/day) led to rapid clinical and radiological resolution. RTX-induced OP requires a high index of suspicion, as its clinical-radiological presentation mimics infectious pneumonia. Early recognition and prompt initiation of corticosteroids are associated with rapid clinical and radiological recovery and may reduce the risk of progression to irreversible pulmonary fibrosis.

## Introduction

Rituximab (RTX) is a genetically engineered chimeric human-mouse monoclonal antibody that specifically binds to the CD20 surface antigen expressed on pre-B and mature B lymphocytes, leading to targeted depletion of CD20+ B cells [1,2]. Since its FDA approval in 1997, it has revolutionized the prognosis of B-cell malignancies [3]. However, its use is associated with a spectrum of drug-induced interstitial lung diseases (DI-ILD), ranging from mild pneumonitis to severe organizing pneumonia (OP) [4,5]. OP is a clinicopathologic pattern characterized by intra-alveolar granulation tissue composed of fibroblasts and myofibroblasts and may arise secondary to infections, autoimmune disease, malignancy, or drug exposure. In drug-associated cases, immune-mediated alveolar epithelial injury and pro-inflammatory cytokine signaling have been proposed as contributory mechanisms. While B-cell depletion predisposes patients to opportunistic infections due to hypogammaglobulinemia, the direct pro-inflammatory pulmonary toxicity of RTX is an increasingly recognized entity that remains underdiagnosed [1,6,7].

## Case presentation

A 71-year-old male with hepatic non-Hodgkin lymphoma (NHL) diagnosed seven years earlier, currently in remission, and receiving maintenance RTX every six months for the preceding four years, presented to the Emergency Department (ED) with a four-week history of worsening dyspnea (Modified Medical Research Council 3), fatigue, anorexia, and a dry cough. His most recent RTX administration had occurred approximately one month before the onset of symptoms. He reported a preceding flu-like syndrome with myalgias. Initial respiratory viral panels (including SARS-CoV-2) were negative. He had recently completed a seven-day course of levofloxacin for suspected pneumonia, with no clinical response.

On admission, the patient was febrile (38.1°C), tachycardic (102 bpm), and hypoxemic (SpO_2_ of 88% on room air). Pulmonary auscultation was unremarkable.

Laboratory findings revealed an elevated C-reactive protein (CRP) level of 4.77 mg/dL (reference <0.50 mg/dL), along with a cholestatic pattern of liver enzyme abnormalities, characterized by aspartate aminotransferase (AST) of 58 U/L (reference <35 U/L), alanine transaminase (ALT) of 54 U/L (reference <45 U/L), alkaline phosphatase (ALP) of 197 U/L (reference 30-120 U/L), and gamma-glutamyl transferase (GGT) of 498 U/L (reference <55 U/L). These liver enzyme abnormalities were newly identified at presentation. They improved during follow-up, although mild persistent abnormalities remained in the context of known metabolic dysfunction-associated steatotic liver disease (MASLD); no direct causal relationship with the pulmonary process could be established. Chest X-ray showed bilateral patchy alveolar infiltrates (Figure 1).

**Figure 1:**
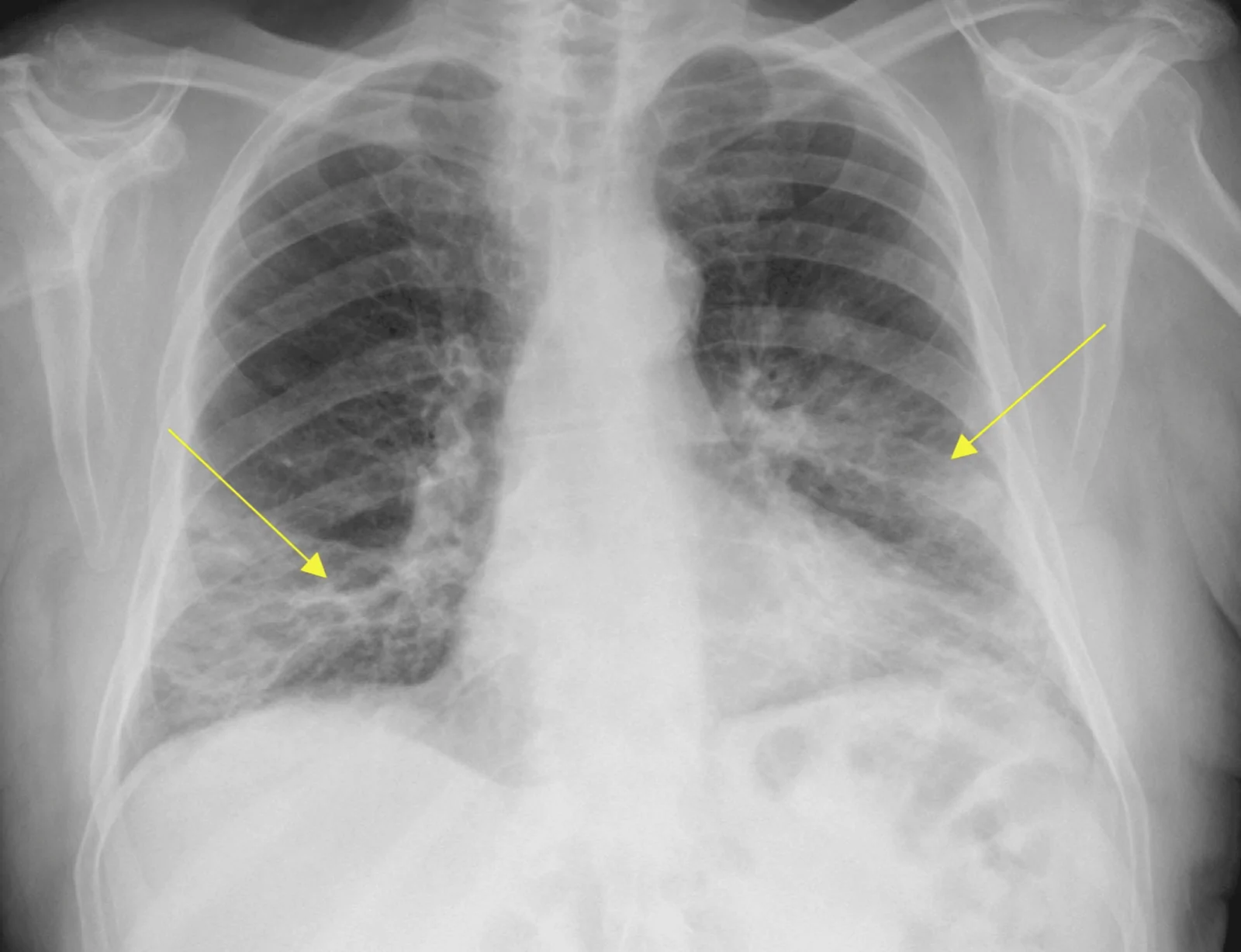
Chest X-ray (anteroposterior view). Patchy bilateral alveolar infiltrates and reticulonodular opacities, predominantly in the lower lung fields, without evidence of pleural effusion or cardiomegaly. The arrows indicate the bilateral patchy alveolar infiltrates.

The patient was hospitalized and started on piperacillin/tazobactam and linezolid. A chest CT scan revealed diffuse bilateral ground-glass opacities (GGO) (Figure 2). Despite transient improvement and discharge, he was readmitted one week later with high-grade fever (39°C), night sweats, and acute hypoxemic respiratory failure.

**Figure 2:**
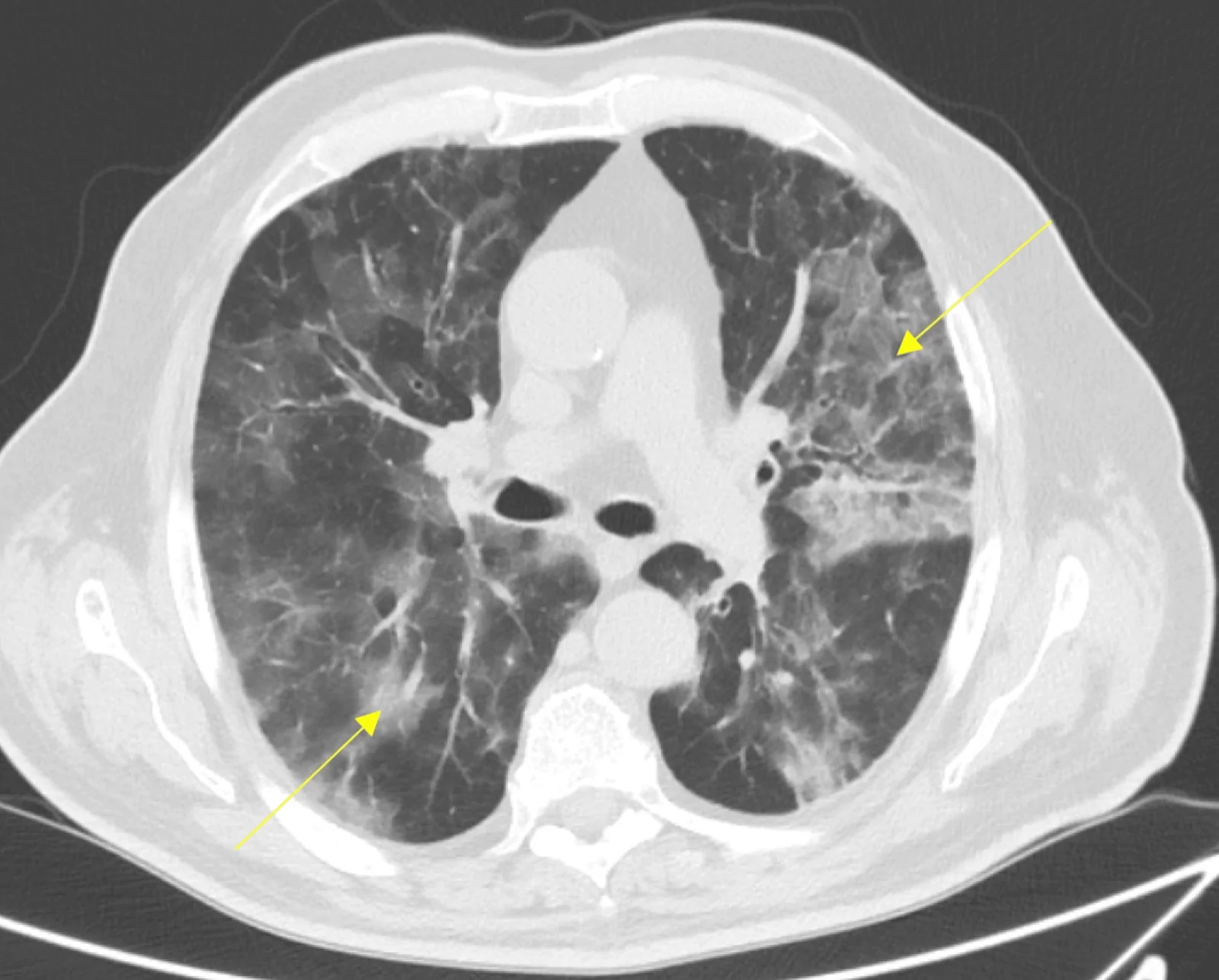
High-resolution computed tomography (HRCT) of the chest. Multiple areas of diffuse "ground-glass" opacities with a geographic and peripheral distribution, associated with early consolidative changes, consistent with an organizing pneumonia (OP) pattern. The arrows indicate representative areas of ground-glass opacity/consolidative change.

To elucidate the etiology of this respiratory failure and persistent fever, further extensive diagnostic investigation was conducted. Microbiological studies, including multiple sets of blood cultures, induced sputum, and bronchoalveolar lavage (BAL) fluid, were negative for aerobic and anaerobic bacteria, *Mycobacterium tuberculosis*, and fungi. Initial serological testing revealed *Coxiella burnetii* IgG titers of 1:160; however, subsequent Phase I and Phase II IgM and IgG antibody assays were confirmed negative, effectively ruling out Q fever.

BAL demonstrated lymphocytosis, while cytology and cultures showed no malignant cells or identifiable pathogens. An autoimmune work-up for secondary causes of organizing pneumonia was negative. Transthoracic echocardiography showed mild mitral regurgitation, preserved global systolic function of both ventricles, and no unequivocal echocardiographic evidence of valvular vegetation, providing no support for a cardiac cause of the respiratory presentation. The main diagnostic investigations and relevant findings are summarized in Table 1.

**Table 1:** Diagnostic work-up and key findings during evaluation of suspected rituximab-induced organizing pneumonia. Abbreviations: ALT, alanine aminotransferase; AST, aspartate aminotransferase; BAL, bronchoalveolar lavage; CRP, C-reactive protein; GGT, gamma-glutamyl transferase; HRCT, high-resolution computed tomography; MASLD, metabolic dysfunction-associated steatotic liver disease; OP, organizing pneumonia; PET/CT, positron emission tomography/computed tomography

Investigation	Key finding
Inflammatory markers	CRP 4.77 mg/dL (reference <0.50 mg/dL)
Liver biochemistry	AST 58 U/L; ALT 54 U/L; alkaline phosphatase 197 U/L; GGT 498 U/L. Newly identified at presentation; subsequently improved, with mild persistent abnormalities in the context of MASLD.
Chest radiograph	Bilateral patchy alveolar infiltrates and reticulonodular opacities, predominantly in the lower lung fields; no pleural effusion or cardiomegaly.
HRCT chest	Diffuse bilateral ground-glass opacities with geographic/peripheral distribution and early consolidative changes, compatible with an OP pattern.
Blood/sputum/BAL microbiology	Negative for aerobic and anaerobic bacteria, *Mycobacterium tuberculosis*, and fungi.
*Coxiella burnetii* serology	Initial IgG 1:160; subsequent Phase I and II IgM/IgG assays negative.
BAL	Lymphocytosis; cytology negative for malignant cells; cultures without identifiable pathogens.
Autoimmune work-up	Negative.
Transthoracic echocardiography	Mild mitral regurgitation; preserved global systolic function of both ventricles; no unequivocal vegetation.
PET/CT	Diffuse metabolic activity corresponding to bilateral pulmonary ground-glass opacities; no evidence of hepatic lymphoma relapse.
Therapeutic response	Rapid apyrexia and oxygen weaning within 48 hours after systemic corticosteroid therapy; near-complete radiological resolution at 3 months.

Imaging played a critical role in the differential diagnosis; a PET-CT scan (Figure 3) was performed, which excluded a relapse of the hepatic NHL but highlighted intense, diffuse metabolic activity within the areas of bilateral ground-glass opacities. Furthermore, flexible bronchoscopy showed a normal endobronchial tree, and BAL cytology and cultures were unremarkable, showing no malignant cells or identifiable pathogens. Given the temporal relationship with RTX administration and the exclusion of infectious and neoplastic causes, a diagnosis of probable RTX-induced organizing pneumonia was considered after exclusion of infectious, neoplastic, autoimmune, and cardiac causes. High-dose corticosteroid therapy with methylprednisolone 250 mg once daily was initiated and maintained for three days. The clinical response was marked, with resolution of fever and successful weaning from oxygen therapy within 48 hours. Corticosteroid therapy was subsequently tapered to 60 mg once daily during hospitalization. The patient was discharged on oral prednisolone 60 mg/day, with a planned dose reduction of 5 mg per week until a maintenance dose of 10 mg/day was reached. Prednisolone 10 mg/day was then continued until outpatient reassessment. At the three-month follow-up, he remained asymptomatic with near-complete radiological resolution (Figure 4). Subsequent clinical follow-up has shown continued stability without recurrence of respiratory symptoms.

**Figure 3:**
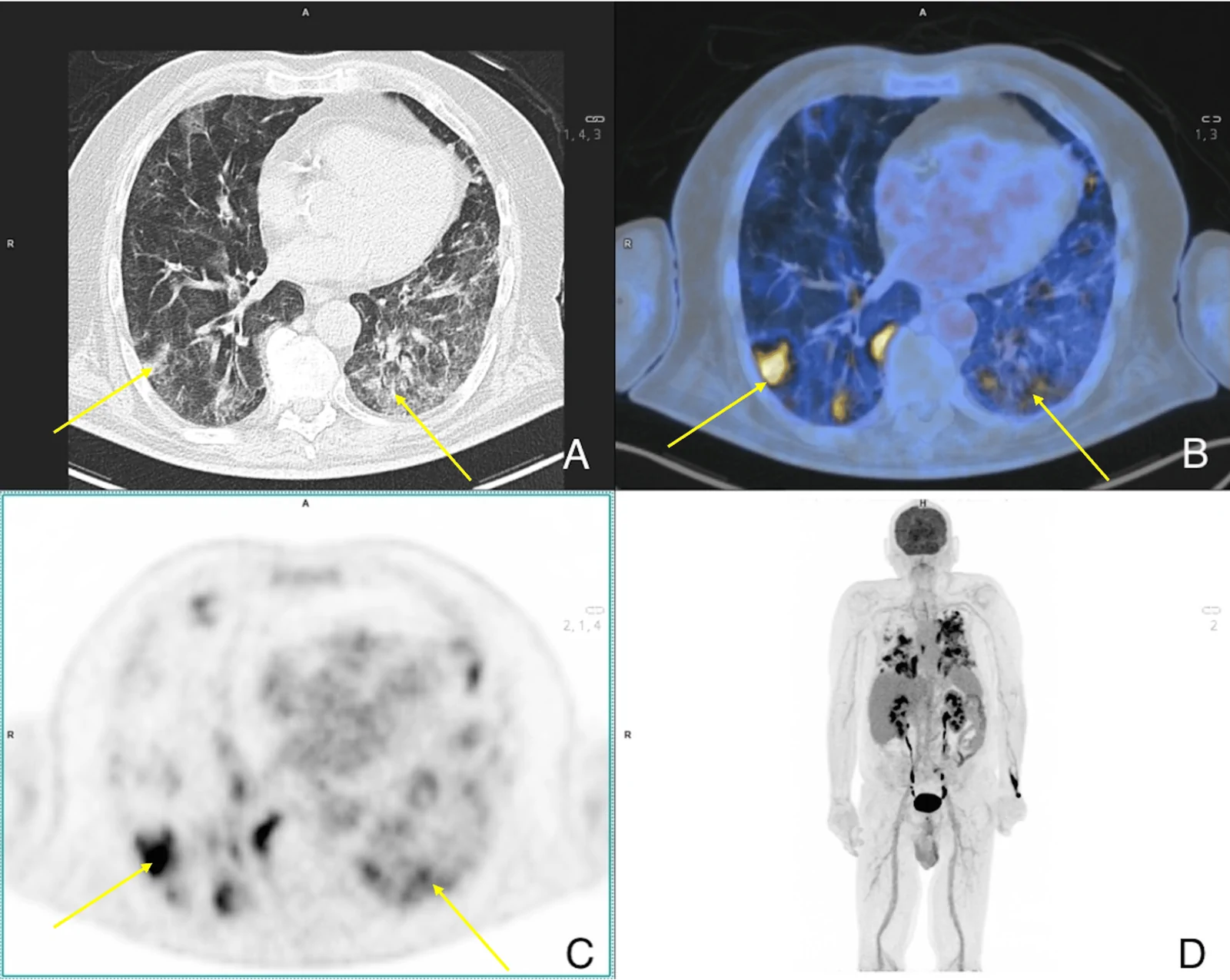
¹⁸F-fluorodeoxyglucose positron emission tomography/computed tomography (¹⁸F-FDG PET/CT). (A) Axial CT (lung window) demonstrating bilateral ground-glass opacities. (B) Axial fused PET/CT image showing intense FDG uptake within the pulmonary infiltrates. (C) Axial PET image confirming diffuse pulmonary hypermetabolism. (D) Whole-body maximum intensity projection (MIP) demonstrating bilateral pulmonary metabolic activity without evidence of hepatic lymphoma relapse.

**Figure 4:**
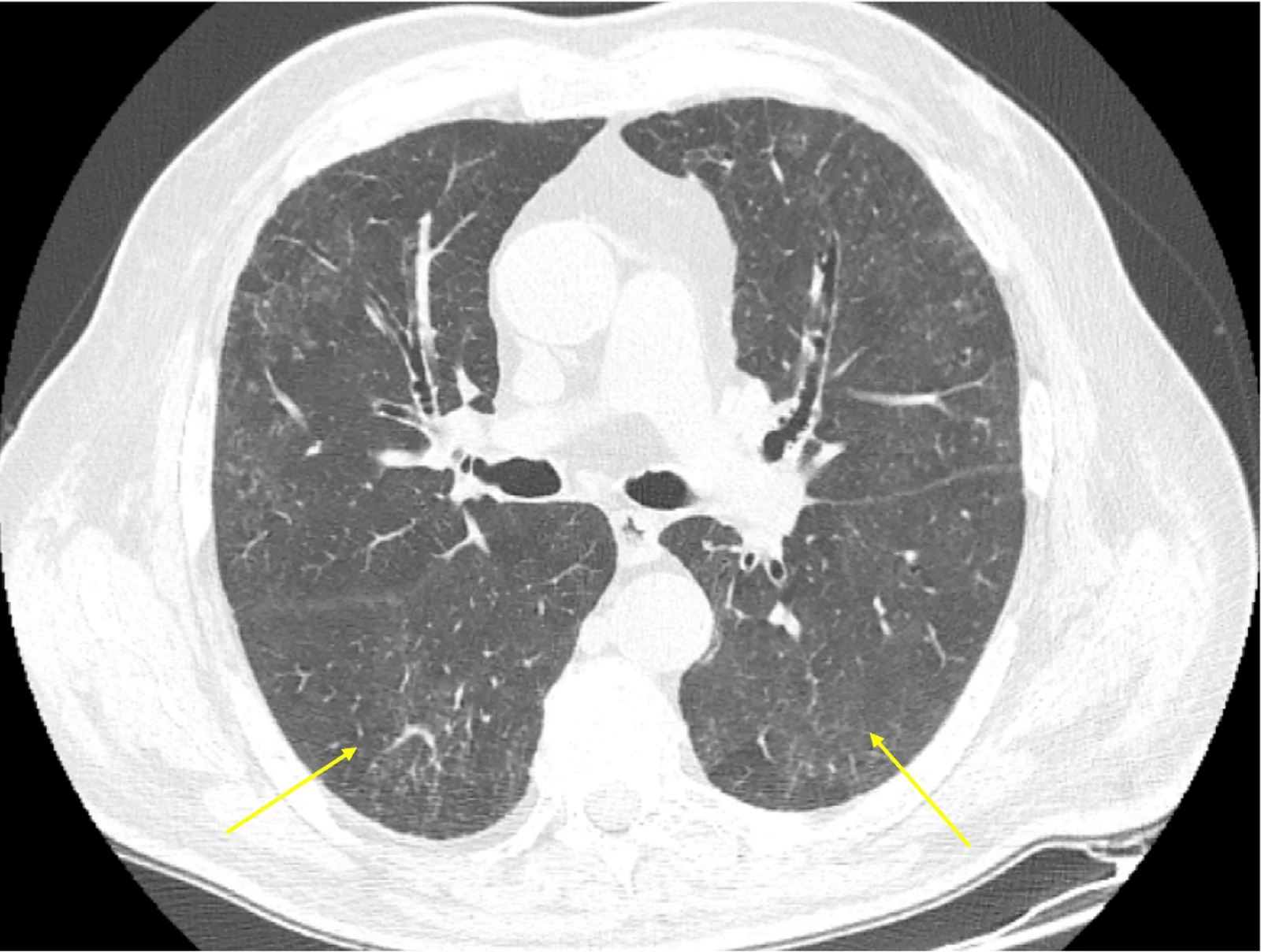
Follow-up chest CT (three months post-corticosteroid therapy). Significant resolution of previous pulmonary opacities, with only faint residual ground-glass areas remaining, confirming an excellent therapeutic response to drug withdrawal and glucocorticoid administration.

## Discussion

The clinical journey of patients treated with RTX is often marked by significant recovery; however, as this case illustrates, there are instances where the treatment itself evolves into a new clinical threat. OP is a clinicopathologic entity characterized by the proliferation of fibroblasts and myofibroblasts within distal airspaces, forming granulation tissue plugs that may extend into alveolar ducts and bronchioles. Clinically, OP is categorized as either cryptogenic, when no underlying cause is identified, or secondary, when associated with conditions such as respiratory infections, autoimmune diseases, malignancy, or drug exposure [4,5]. The typical presentation includes a nonproductive cough, low-grade fever, malaise, dyspnea, and weight loss, often following a flu-like illness. This nonspecific clinical picture frequently delays diagnosis, as patients are commonly treated with multiple courses of antibiotics before OP is considered.

In the present case, the preceding flu-like syndrome and the transient clinical improvement during antibacterial therapy required careful consideration of infection as a potential trigger or alternative diagnosis. However, repeated microbiological studies, BAL cultures, serological reassessment, and the subsequent clinical course did not identify an active infectious etiology. A preceding infection therefore cannot be completely excluded as a possible trigger for secondary OP, but the temporal relationship with RTX exposure, exclusion of other major causes, and prompt corticosteroid response supported RTX-associated OP as the most likely diagnosis.

Radiologically, OP is characterized by patchy, lower-lobe-predominant consolidation and ground-glass opacities, often with a peribronchovascular or subpleural distribution [1,8]. While BAL usually reveals lymphocytosis and helps exclude infectious causes, as was observed in this patient, a lung biopsy remains the gold standard for diagnostic confirmation.

A notable limitation of the present report was the absence of histopathological examination, as the patient strongly refused a lung biopsy despite medical recommendation. This refusal, combined with the unfavorable progression after initial antibiotic therapy, the characteristic pattern of lesions on chest CT, and the known history of RTX exposure, justified the decision to initiate systemic corticosteroid therapy with methylprednisolone. It is important to emphasize that, while surgical lung biopsy via thoracoscopy or open procedure is the definitive diagnostic tool, it may carry a high risk in critically ill patients, leading clinicians to rely on a diagnosis of exclusion and response to treatment [5].

Cases of OP associated with RTX therapy are exceedingly uncommon and are mostly documented as individual case reports [9,4]. Typically, RTX's adverse effects are transient, cytokine-mediated hypersensitivity reactions occurring during initial infusions. Nevertheless, emerging evidence suggests that repeated administration may trigger OP in susceptible individuals [6,10]. The underlying mechanisms are thought to involve complement activation and the release of proinflammatory cytokines, such as TNF-α, IL-6, and IL-8. Additionally, cellular lysis may release peptides that stimulate dendritic cell maturation and cytotoxic T-cell activation, resulting in delayed immune-mediated injury of the alveolar epithelium [2,4]. This cascade explains the temporal association between OP and cumulative RTX administration observed in this case. In nearly all reported instances, the discontinuation of RTX and the initiation of corticosteroid therapy led to marked clinical and radiological improvement, reinforcing the need for early recognition to ensure complete recovery [1,9].

## Conclusions

RTX-induced OP represents a significant clinical challenge, requiring a high index of diagnostic suspicion, as its clinical and radiological manifestations frequently mimic common infectious processes in immunocompromised patients.

This case highlights the importance of considering drug-induced pulmonary toxicity as an important differential diagnosis in patients undergoing rituximab therapy who present with new interstitial infiltrates and fever, particularly after the exclusion of infectious etiologies and recurrence of the underlying malignancy. Early recognition of this entity is crucial, allowing for the discontinuation of the suspected causative agent when clinically appropriate and the initiation of systemic corticosteroid therapy, which remains the cornerstone of management and typically yields rapid clinical and radiological improvement. The favorable outcome observed in this patient underscores that prompt recognition and management may be associated with substantial clinical recovery and may reduce the risk of progression to irreversible pulmonary fibrosis.
